# Development and validation of an educational comic book on healthy eating in early childhood

**DOI:** 10.1017/jns.2025.10072

**Published:** 2026-02-05

**Authors:** Cristiano Carvalho Soares, Luciane Zanin, Marcelo Sperandio, Flávia Martão Flório

**Affiliations:** 1 Faculdade São Leopoldo Mandic, Campinas, SP, Brazil; 2 Universidade Federal de Lavras (UFLA), Lavras, MG, Brazil

**Keywords:** Child, Comic book, Food and nutrition education, Food and nutritional health promotion, Health education, Preschool, Validation study

## Abstract

The objective of this study was to develop and validate an educational comic book designed to promote healthy eating among caregivers of young children. The study was conducted in four phases: (1) literature review and script development; (2) creation of the initial version of the comic book, including illustrations, layout and design, and calculation of the Flesch Readability Index (FI); (3) expert validation of the initial version and calculation of the Content Validity Index (CVI); and (4) adaptation of the comic book based on expert suggestions, recalculation of the FI, and pilot testing (CVI) with a lay population. A total of 64 volunteers participated in the validation process, including 14 expert judges and 50 caregivers responsible for feeding children aged 0 to 5 years. Statistical analysis included descriptive measures and inferential testing using the Wilcoxon signed-rank test. The FI score for the initial version was 85.0%, indicating a reading level classified as “easy to understand.” After expert evaluation, the CVI reached 94%, reflecting high agreement among participants. In the revised version, the FI remained high at 84.7%, reinforcing the “easy to understand” reading level, while the CVI increased to 98% following the pilot test, demonstrating strong consensus among participants. A significant improvement in knowledge regarding healthy eating was observed after reading the comic book (*p* < 0.05). The comic book was validated for appearance, content and readability, showing a positive impact on caregivers’ knowledge about healthy eating practices. It represents an accessible and effective resource that can be integrated into community-based nutrition education programmes.

## Introduction

Promoting healthy eating in children is a key public health priority, given its influence not only on lifelong eating behaviors but also on the predisposition to various long-term morbidities, including obesity, ischemic heart disease, and dental caries.^(1,2)^ Within this context, the family environment plays a fundamental role in the implementation of appropriate dietary strategies, given that family members are central to shaping healthy habits. Evidence consistently shows that early adoption of adequate dietary practices leads to more favorable long-term outcomes, and the family’s eating pattern should therefore be regarded as a modifiable factor with the potential to influence health throughout life.^(3,4)^

Healthy eating from pregnancy onward is essential to ensure proper child growth and development. During the first 1,000 days of life, from conception to two years of age, adequate maternal and infant nutrition helps reduce cardiometabolic risk and future obesity while supporting cognitive development and human capital formation.^(5–7)^ Adequate intake of macro- and micronutrients plays a vital role in this process: folic acid prevents neural tube defects, iron supports brain development, and docosahexaenoic acid (DHA) deficiency is linked to premature birth.^(8–10)^ Exclusive breastfeeding up to 6 months, followed by the gradual introduction of in natura or minimally processed foods, is fundamental for ensuring healthy growth and balanced microbiota composition, thereby reducing the risk of noncommunicable diseases.^(11–17)^

Educational technologies are increasingly used to disseminate health information effectively.^(18)^ Within nutrition education, several strategies have shown promising outcomes, such as comic books, illustrated brochures, and visual media that enhance engagement and retention of information.^(19–23)^ The use of comics in health education has been validated across multiple contexts, proving to be an innovative and playful yet effective method for conveying complex information and promoting behavioral change.^(24–32)^

The *Playing and Learning About Health* initiative aims to provide lay audiences with accessible educational materials. This study builds on that framework, focusing on the development and validation of a comic book designed to promote healthy eating among caregivers of children aged 0 to 5 years, as well as to assess its impact on participants’ knowledge.

## Methods

This study involves the development of an educational technology. It was conducted in accordance with the ethical standards outlined in Resolution 466/2012 of the National Health Council of the Ministry of Health and was approved by the Research Ethics Committee (CAAE: 55114821.6.0000.5374). Financial support was provided by the São Paulo Research Foundation (FAPESP; Grant number: 2022/02599-6), which funded only the research-related costs. The funding agency had no role in the design of the study, data collection, analysis, interpretation, manuscript preparation, or the decision to submit the article for publication.

The validation of the comic book involved an invitation extended to 31 expert judges and 62 representatives of the target audience, who were caregivers responsible for feeding children aged 0 to 5 years. The selection of expert judges was based on curriculum analyses available on the Lattes Platform (http://lattes.cnpq.br/), an integrated database of individual researchers and Brazilian institutions, supervised by the National Council for Scientific and Technological Development (CNPq). Inclusion criteria required that candidates meet at least two of the following requirements: a minimum of 10 years’ experience in child-focused health promotion or prevention activities; scientific publications related to food health and/or childhood obesity; participation in the development and validation of educational materials; and possession of a master’s or doctoral degree with scientific output in the fields of nutrition, collective health or pediatrics. Representatives of the target audience were recruited by convenience sampling in the waiting room of a private pediatric clinic in Lavras, Minas Gerais (MG). To be eligible, participants had to be responsible for feeding at least one child aged 0 to 5 years.

The study was conducted in four phases:

– Script creation:

The story script was developed after reviewing the literature and consulting guidelines published by the Brazilian Society of Pediatrics^(33)^ and the Ministry of Health.^(34)^ The narrative follows a character who, after a typical day, wakes up the next morning, looks into the mirror, and finds that her appearance has changed. Seeking answers, she visits a friend, who helps her recall the healthy eating practices her family has followed. The script emphasizes key aspects of food health, including maternal nutrition during pregnancy,^(35,36)^ exclusive breastfeeding until six months of age,^(37,38)^ the introduction of all food groups during complementary feeding,^(39,40)^ the exclusion of processed and ultra-processed foods,^(41–43)^ and the importance of healthy snacks during the school year.^(44,45)^

– Preparation of the initial version of the comic book (illustrations, layout, design):

Based on the script, original illustrations were developed and characters were created. Page formatting, layout, and design were executed using Adobe Photoshop® and Adobe InDesign®, in collaboration with an experienced graphic designer. Content development followed key criteria related to substance, structure and organization, language, layout, design, cultural sensitivity, and contextual appropriateness for the intended audience. The characters were modeled after those used in previous publications from the *Playing and Learning About Health* project. To ensure clarity and accessibility, inclusive and respectful language was used to facilitate understanding and emphasize the roles of both individuals and families in promoting healthy eating practices.^(46)^ During this phase, the Flesch Readability Index (FI) was calculated to ensure a minimum threshold of 70%, and classified the material as reasonably easy to very easy to understand.^(24,46)^

– Validation of the initial version:

Expert judges were provided with a printed copy of the comic book along with a questionnaire containing eight items to gather sociodemographic data. After a careful and critical reading of the material, they completed an evaluation questionnaire,^(47)^ which was organized into three sections: adequacy (5 items), structure (8 items), and relevance (5 items) of the comic book. Responses were recorded using a four-point Likert scale: Totally Adequate (TA), Adequate (A), Partially Adequate (PA), and Inadequate (I). Space was provided for the judges to justify their ratings and offer suggestions for improvement.

Based on the judges’ responses, the Content Validity Index (CVI) was calculated for each item (I-CVI), for each block (S-CVI/AVE Block), and globally (S-CVI/AVE Global). The CVI measures the relevance and representativeness of each element in a research instrument, with values ranging from 0 to 1. The calculation involved summing the number of responses rated as “Adequate” and “Totally Adequate,” dividing by the total number of responses, and multiplying by 100. The minimum acceptable threshold defined for this study was 80%.^(25,26,48)^

– Preparation of the revised version of the comic book and pilot test:

Following the revisions to the comic book based on the expert judges’ suggestions, the FI was recalculated, and the final version of the material was completed. A pilot test was then conducted with representatives of the target audience. Before reading the comic book, participants completed a questionnaire comprising four sociodemographic questions and six questions assessing knowledge of practices related to healthy eating. This questionnaire – developed by the researchers and reviewed by 11 pediatricians and nutritionists – addressed the following topics: characteristics of a healthy diet^(49,50)^; recommendations for exclusive breastfeeding^(51,52)^; strategies for introducing complementary feeding^(53,54)^; characteristics of healthy snacks for children^(55)^; eating behaviors in children over 2 years of age^(56)^; and appropriate snack choices during the school year.^(56)^

The printed comic books were distributed, and after being made available for 15 days, participants completed an evaluation questionnaire regarding the material. The questionnaire was divided into 4 blocks: reading process (3 questions), content (6 questions), visual (5 questions), and characters (3 questions), using a valuation scale from “Totally Adequate” (TA) to “Inadequate” (I). Additionally, five questions addressed the organization, writing style, appearance, narrative motivation, and overall usefulness of the comic book.^(25,26)^ At this time, the knowledge questionnaire was also reapplied. The CVI for version 2 of the material was calculated at the item level (I-CVI), block level (S-CVI/AVE Block), and globally (S-CVI/UA Global).

The educational comic book developed and validated in this study is entitled *Dentitos – Playing and Learning About Health: Food Health*. On the inside cover, the Playing and Learning About Health initiative is introduced, along with its objective of using educational technologies to reach the target audience. The body of the publication consists of a comic strip, while the final section presents a timeline highlighting the key recommendations for food health in children aged 0 to 5 years, as well as thematic activities. The material was registered with the Brazilian Book Chamber (CBL) under ISBN: 978-65-86718-61-4 (Portuguese and English versions) and comprises a cover, back cover, and 16 pages, formatted in a standard size of 21 cm high by 14.8 cm wide. The final version of the comic book is available for free download in both Portuguese and English^1^.

For each version of the comic book, an exploratory statistical analysis was conducted using Microsoft Office Word, assessing components of the material, including the presentation and body of the comic strip. The number of pages, words, characters (with and without spaces), paragraphs, and lines – typed using single spacing – were recorded. Descriptive and exploratory analyses were also performed to characterize the profiles of the expert judges and members of the target audience, based on absolute and relative frequencies.

The FI was calculated for both versions (01 and 02) to evaluate the readability level of the text, based on a scale from 0 to 100. Higher FI values indicate easier reading and suggest that the material requires a lower level of formal education for comprehension by a lay audience.^(24)^ CVIs were then estimated at the item level (I-CVI) for each block of the evaluation questionnaire completed by expert judges (objectives, structure, and relevance) and by the target audience (content, audiovisual elements, and characters). The CVI quantifies the proportion of evaluators who consider each item relevant or representative.^(46)^ For this purpose, responses were grouped into two categories: “relevant/representative” (sum of responses rated “Totally Adequate” and “Adequate”) and “needs correction” (sum of responses rated “Partially Adequate” and “Inadequate”). Subsequently, CVIs were calculated at the block level (S-CVI/AVE), representing the average of the I-CVIs within each block, as well as the global level.

CVIs were also estimated at the scale level (S-CVI/UA), which measures the proportion of items rated as positive by each judge or layperson, along with the global average. In the present study, a minimum CVI of 0.70 (70%) was considered acceptable,^(57)^ while a more stringent threshold of 80% was established to ensure the validity of the material.^(24–26,58)^ To compare scores on the knowledge questions before and after reading the comic book, the paired Wilcoxon test was applied. In addition, the absolute and relative frequencies of participants were calculated based on the change in the total score following the intervention. All analyses were conducted using R software, with the significance level set at 5%.

## Results

Table 1 presents the profile of the expert judges and the representatives of the target audience who participated in the study. Among the 31 expert judges invited, 14 agreed to evaluate the initial version of the comic book. Most of the judges were women (76.8%), and over 51 years of age (57.1%). All held *stricto sensu* graduate degrees and had more than 10 years of professional experience. Regarding the target audience, the majority of the participants were women (82%), aged 40 years or younger (68%), and had completed college (76.0%).

**Table 1.** Sociodemographic characteristics of expert judges (*n* = 14) and the representatives of the target audience (*n* = 50)

Variable	Category	Frequency (%)
*Expert judges*		
Sex	Female	11 (78.6%)
	Male	3 (21.4%)
Age group	31–40 years	3 (21.4%)
	41–50 years	3 (21.4%)
	≥51 years	8 (57.1%)
Do you have children?	No	1 (7.1%)
	Yes, I currently have school-age children	5 (35.7%)
	Yes, but my children are older now	8 (57.1%)
^1^Expertise	>10 years of professional experience	14 (100.0%)
	MSc or PhD	14 (100.0%)
	Scientific publications on child obesity	5 (35.7%)
	Construction and validation of educational materials	2 (14.2%)
Sector of work	Public and private	4 (23.1%)
	Private	2 (14.2%)
	Public	7 (50.0%)
	No information	1 (7.1%)
*Representatives of the target audience*		
Sex	Female	41 (82.0%)
	Male	9 (18.0%)
Age group	≤30 years	6 (12.0%)
	31–40 years	28 (56.0%)
	41–50 years	13 (26.0%)
	≥51 years	3 (6.0%)
Schooling	Partial secondary education	1 (2.0%)
	Complete secondary education	6 (12.0%)
	Incomplete college education	5 (10.0%)
	Complete college education	38 (76.0%)
Number of children	1	21 (42.0%)
	2	24 (48.0%)
	3	3 (6.0%)
	No information	2 (4.0%)

Table 2 presents the exploratory analysis of statistics generated using Microsoft Office Word, as well as the calculation of the FI and its corresponding reading ease classification. Following the expert evaluation of the initial version, modifications were made – primarily an increase in the amount of text in the introductory section and a reduction in the text within the comic strip itself. These adjustments did not affect the overall readability of the material. In both versions, the F1 scores remained between 80 and 90, classifying the content as “easy to understand.”

**Table 2.** Exploratory statistics generated by Microsoft Office Word, Flesch Readability Index (FI), and readability classification for each section of the comic book

	Presentation		Comic book content	
	Initial version	Revised version	Initial version	Revised version
Pages	1	1	2	3
Words	73	76	705	686
Characters (no spaces)	342	353	3365	3293
Characters (with space)	412	426	4017	3918
Paragraphs	3	3	56	63
Lines	5	5	56	66
FI	82.20	80.16	85.45	84.68
Readability	Easy to understand text (80–90)			

Table 3 presents the CVIs of the initial version of the comic book, as rated by expert judges. These include item-level indices (I-CVI), block-level averages (S-CVI/AVE Block), and the overall index (S-CVI/AVE Global). The global CVI was 0.94 (94%), a value equivalent to the indices obtained for the “Objectives,” “Structure and Presentation,” and “Relevance” blocks. These values indicate excellent agreement in the evaluations performed by the judges. Furthermore, all items evaluated in each block presented CVIs equal to or greater than 0.86. Most of the expert judges’ suggestions were incorporated into the revised version. These included rephrasing and condensing certain dialogues and narrative passages, as well as refining terminology to make technical content more accessible to lay readers. Modifications were also made to broaden the portrayal of caregiving responsibilities, emphasizing the role of all caregivers – not just mothers. In addition, adjustments were made to the positioning of selected illustrations for improved visual coherence. The main focus of the comic book, originally centered on childhood obesity, was expanded to encompass general dietary health. This change reflected the broader scope of the content and acknowledged that obesity, as a multifactorial condition, could not be addressed comprehensively within the format of the comic book.

**Table 3.** Content validity indices by item, block, and overall, according to expert judges – Initial version of the comic book (*n* = 14)

Item evaluated	Relevance	Needs minor corrections	CVI
	FA + A	PA + I	
**Block 1–Objectives**	**–**	**–**	–
Consistent information	13	1	0.93
Content importance	14	0	1.00
Incentive to change behavior	13	1	0.93
Circulation in the scientific community	13	1	0.93
Meets the objectives of institutions	13	1	0.93
**S-CVI/AVE–Block 1**	**–**	**–**	**0.94**
**Block 2–Structure and presentation**	**–**	**–**	–
Adaptation to the target audience	13	1	0.93
Clarity and objectivity of messages	12	2	0.86
Scientific suitability	14	0	1.00
Adequacy to sociocultural level	14	0	1.00
Logical sequence of content	13	1	0.93
Structuring of information	12	2	0.86
Grammatical agreement and spelling	14	0	1.00
Writing style	13	1	0.93
**S- CVI/AVE–Block 2**	**–**	**–**	**0.94**
**Block 3–Relevance**	**–**	**–**	**–**
Key aspects are reinforced	14	0	1.00
Generalization and transfer of learning	13	1	0.93
Allows construction of knowledge	13	1	0.93
Subject material is needed by the target audience	12	1	0.92
Any professional can use it	13	1	0.93
**S- CVI/AVE–Block 3**	**–**	**–**	**0.94**
**S- CVI/AVE–Overall**	**–**	**–**	**0.94**

A, Adequate; FA, Fully Adequate; PA, Partially Adequate; I, Inadequate; CVI, Content Validity Index; S-CVI/AVE, Scale-Level Content Validity Index (average calculation method).

After implementing these changes, the revised version of the comic book was submitted for evaluation by the target audience. The CVIs derived from the responses are shown in Table 4. The average scale-level CVI (S-CVI/UA) was 0.98, exceeding the established minimum threshold of 0.80. The CVI was 0.99 (99%) for the “Content” block, 0.97 (97%) for the “Visual” block, and 0.99 (99%) for the “Characters” block. The lowest I-CVI observed was 0.94 (94%), indicating strong agreement among participants regarding the quality and relevance of the material.

**Table 4.** Content validity indices by item, block, and overall, according to the general public – Revised version of the comic book (*n* = 50)

Item evaluated	Relevance	Needs minor corrections	CVI
	FA + A	PA + I	
**Block 1–Content**	–	–	–
Consistent information/content	50	0	1.00
Clear and understandable information/content	48	2	0.96
Inviting content presentation	48	2	0.96
Appropriate for circulation in the scientific community	50	0	1.00
Meets project aims	50	0	1.00
Logical content sequence	50	0	1.00
**S-CVI/AVE–Block 1**	**–**	**–**	**0.99**
**Block 2–Visual Presentation**	–	–	–
Visually adequate and supports comprehension	48	2	0.96
Images are consistent with the content	50	0	1.00
Appropriate scenario	50	0	1.00
Illustrations are consistent with the content	48	2	0.96
Appropriate colors and framing of images	47	3	0.94
**S- CVI/AVE–Block 2**	**–**	**–**	**0.97**
**Block 3–Characters**	–	–	–
Characters speak clearly	49	1	0.98
Characters present themselves coherently	49	1	0.98
Speech is coherent and reflects reality	50	0	1.00
**S- CVI/AVE–Block 3**	**–**	**–**	**0.99**
**S- CVI/AVE–Overall**	–	–	**0.98**

A, Adequate; FA, Fully Adequate; PA, Partially Adequate; I, Inadequate; CVI, Content Validity Index; S-CVI/AVE (Scale-level Content Validity Index, average calculation method).

The descriptive analysis of the overall evaluation revealed a highly favorable reception of the comic book. A total of 98% (*n* = 49) of readers considered the illustrative cover to be visually appealing and conducive to engagement, and 98% (*n* = 49) found the text presentation interesting. All participants (100%, *n* = 50) agreed that the illustrations enhanced their understanding of the narrative in an imaginative and playful manner. Additionally, 98% (*n* = 49) reported feeling motivated to read the story to its conclusion, and 100% (*n* = 50) believed the comic book would contribute to health education efforts for the general population.

Table 5 shows that reading the comic book led to an increase in the number of correct responses on the knowledge assessment instrument concerning healthy eating practices for children aged 0 to 5 years.

**Table 5.** Analysis of participants’ knowledge scores before and after reading the comic book (*n* = 50)

Question topic		Score			*p*-value
	Before		After		
	Median (^1^IQR)	Min-Max^2^	Median (^1^IQR)	Min-Max	
Healthy eating	1.0 (1.0; 1.0)	1–1	1.0 (1.0; 1.0)	1–1	^3^–
Exclusive breastfeeding	2.0 (2.0; 2.0)	0–2	2.0 (2.0; 2.0)	0–2	0.2945
Complementary feeding	1.0 (1.0; 1.0)	0–1	1.0 (1.0; 1.0)	0–1	0.3173
Snacks between meals (children > 1 year)	2.0 (2.0; 2.0)	1–2	2.0 (2.0; 2.0)	1–2	0.1422
Feeding children > 2 years	3.0 (3.0; 3.0)	0–3	3.0 (3.0; 3.0)	0–3	0.1088
School feeding (children ≤ 5 years)	2.0 (2.0; 2.0)	0–2	2.0 (2.0; 2.0)	0–2	0.7794
Total	11.0 (10.0; 11.0)	6–11	11.0 (10.0; 11.0)	7–11	0.0238

^1^IQR: Interquartile Range (Q1; Q3). ^3^ *p*-value could not be calculated due to lack of variation among responses; all had a score of 1 both before and after reading.

Table 6 presents the descriptive analysis of responses to the knowledge assessment questionnaire administered before and after the intervention. While participants already demonstrated satisfactory knowledge on the general concept of healthy eating during the pre-test (100% correct), improvements were observed in several specific areas after reading the comic book. Notably, correct responses regarding the recommended duration of exclusive breastfeeding and complementary feeding increased from 86% to 94%, the ability to identify appropriate snacks between meals for children over one year of age rose from 90% to 98%, and knowledge of dietary behavior in children over two years of age improved from 86% to 92%. Additionally, marked gains were observed in the proportion of participants who achieved maximum scores on items related to the initiation and care of complementary feeding (from 96% to 98%), and school feeding for children up to five years of age (from 76% to 78%).

**Table 6.**
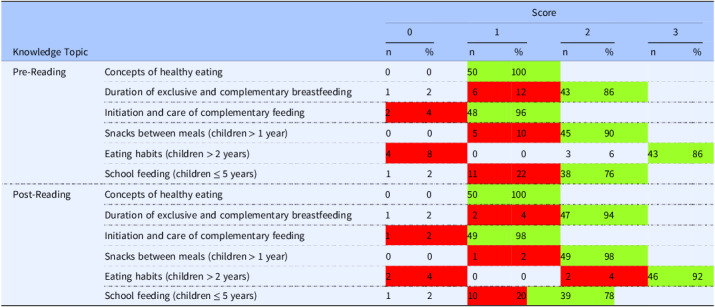
Descriptive analysis of the questionnaire responses on knowledge of healthy eating in children (*n* = 50)

Legend: green – highest frequency of responses, red – second highest frequency of responses.

## Discussion

The use of comic books in health education has proven effective across a range of contexts.^(19,26,59)^ In the present study, the validated comic book emerged as a valuable resource for promoting healthy eating practices in children aged 0 to 5 years. It achieved high CVI scores and demonstrated strong acceptance by the target audience. CVI values exceeded 80% in both stages of evaluation, surpassing the minimum acceptable threshold of 70%^(24)^ and reaching the ideal benchmark of 85%.^(57)^ These findings indicate that the material was considered representative and relevant by expert judges, while also being clearly understood and positively received by the lay audience.

The evaluation of readability is vital to prevent learning limitations related to low educational attainment.^(18)^ The classification of the comic book in terms of ease of reading and comprehension^(24)^ confirmed its suitability even for individuals with limited formal education. Expert judges highlighted the authors’ attention toward minimizing the amount of text and maximizing the use of images, thereby enhancing accessibility to information. This emphasis is especially important considering that limited knowledge about healthy eating in childhood can lead to adverse outcomes in both the short^(60,61)^ and long term.^(62)^

Food preferences and dietary habits begin to form during the earliest years of life.^(63)^ Evidence shows that the attitude and behaviors of caregivers are key to ensuring adequate nutritional health in childhood.^(64–67)^ Educational strategies directed at caregivers demonstrate effectiveness in increasing knowledge about infant feeding,^(68)^ which is associated with both the encouragement of healthy food consumption by children^(63,69)^ and how caregivers engage in nutritional education.^(70)^

In this study, the target audience comprised adults responsible for feeding children under five years of age, recruited from the waiting room of a private pediatric clinic. Most participants had completed college, which may explain the high baseline knowledge observed.^(71)^ This characteristic likely contributed to the absence of statistically significant differences in some comparisons, suggesting a possible ceiling effect that should be considered when interpreting the Wilcoxon test results. Such effects are common in educational and behavioral interventions when participants exhibit high baseline knowledge or favorable attitudes before exposure, which limits the observable margin for improvement. Similar outcomes have been reported in studies using comics and visual narratives in health education, where participants with higher literacy or prior familiarity with the topic demonstrated strong baseline comprehension, resulting in small post-intervention gains despite high engagement and acceptance of the tool.^(14,31)^

Although education does not directly determine income, it is often associated with greater job opportunities and financial stability. Income also shapes the home food environment: more affluent families often provide conditions favorable to healthy food choices, while those with fewer resources may lack adequate storage, preparation space, or access to markets offering fresh produce.^(63,70,72)^ In Brazil, maternal education has been shown to influence the quality of a child’s diet; mothers with lower education levels or younger age often provide less dietary diversity and poorer overall nutrition.^(73)^

Despite the predominantly high educational level of the sample, composed mainly of caregivers recruited from a private pediatric clinic, the comic book had a positive impact on participants’ knowledge. However, this profile may limit the generalizability of the findings to populations with different socioeconomic and literacy backgrounds. Future studies should include participants from more socioeconomically diverse contexts and assess the applicability of this educational tool in school- and community-based nutrition education programs aimed at promoting healthy eating habits from early childhood.^(74)^

Overall, participants demonstrated awareness of the fundamental aspects of a healthy diet, including the importance of a balanced intake rich in fruits, vegetables, and legumes consumed daily. Targeted interventions, such as promoting family meals and educating parents, have been shown to increase fruit and vegetable consumption among children.^(72)^ A dietary pattern that incorporates a variety of food groups ensures the intake of essential nutrients needed for proper growth, cognitive development, and immune system support in early childhood.^(73)^ Participants also recognized the importance of exclusive breastfeeding for the first six months of life, followed by the introduction of complementary foods, including cereals, tubers, legumes, vegetables, meats, eggs, and fruits. Continued breastfeeding should be encouraged, with emphasis on both its duration and the critical role of exclusivity during the first six months, since this establishes a foundation for long-term health and development.^(75)^

Regarding snacks between meals for children over one year of age, respondents recognized that these should consist of light, nutritious options such as fruits, bread rolls with cheese, and milk. Regarding 100% fruit juice – that is, juice without sugars or preservatives – there is still controversy in the literature, since the addition of such ingredients is a common habit in many cultures around the world. Some studies indicate that moderate consumption of pure fruit juice does not increase the risk of obesity,^(76,77)^ and suggest that it may complement the intake of *in natura* fruits, though it should not replace them entirely.^(77)^ However, there is growing evidence that excessive consumption of fruit juice can lead to increased intake of calories and sugars.^(78)^ The consumption of yogurt is somewhat less controversial, since it is generally associated with nutritional benefits, particularly due to its high calcium and protein content.^(79,80)^ Nonetheless, the sugar content of commercially available yogurts varies widely, and excessive intake may negatively affect a child’s health. For this reason, the recommendation is to opt for natural yogurts or, at least, low-sugar alternatives.^(81)^

Analysis of the issue regarding school snacks revealed that, even after reading the comic book, some participants continued to view processed foods as acceptable options. This perception may reflect the growing time pressures faced by families – particularly mothers – who often balance professional obligations with household responsibilities.^(82,83)^ In the revised version of the comic book, the topic of shared responsibility for children’s nutrition was addressed, aiming to challenge and reshape cultural paradigms in societies where such duties are disproportionately assumed by women.^(84)^ Although equitable distribution of caregiving duties is ideal, the high proportion of female participants in this study indirectly reflects a reality in which most children attending medical appointments are accompanied by their mothers.

In regard to school snacks, it is important to note that in Brazil, Federal Law No. 11.947, of July 16, 2009, guarantees the right to healthy food in public schools, requiring that snacks be prepared under the supervision of nutritionists. However, this legislation does not apply to private institutions. A persistent challenge involves the lunchboxes prepared by families – especially mothers – for their children attending private schools, where the nutritional quality of food often differs from that offered in public schools.^(85)^ International literature also highlights differences between school systems. In some countries, private schools exhibit better nutritional practices,^(86)^ and disparities have also been observed in the types of food options sold in retail outlets surrounding public and private schools.^(87)^

In addition to the comic strip itself, the educational material also included a timeline summarizing key milestones in healthy eating, such as exclusive breastfeeding up to six months^(51)^ and the timely introduction of complementary foods.^(13)^ This timeline reinforces the importance of establishing appropriate feeding practices from gestation through early childhood, consistent with current literature and public health recommendations.^(89)^

The use of comic books in health education has proven effective across various contexts. In the present study, the validated comic book emerged as a valuable resource for promoting healthy eating practices in children aged 0 to 5 years, achieving high CVI scores and strong acceptance by the target audience. CVI values exceeded the ideal benchmark of 85%, confirming the material’s relevance and clarity.

Importantly, the evaluation of readability ensured that the material was accessible even to individuals with limited formal education, enhancing its public health applicability. However, it is important to acknowledge that the sample predominantly comprised caregivers with higher educational attainment, recruited from a private pediatric clinic, which may limit the generalizability of the findings to populations with lower literacy levels or different socioeconomic backgrounds. Future research should evaluate the impact of this educational tool in more diverse populations.

## Conclusion

The educational comic book was validated for appearance, content, and readability, demonstrating a positive impact on caregivers’ knowledge regarding healthy eating practices for children aged 0 to 5 years. Given its accessibility and effectiveness, the comic book may serve as a scalable tool in community-based nutrition education programs.

## Footnotes

1
https://drive.google.com/drive/folders/1uGmenemUrtAYGjGVq10Zoy26It0e4Q7p?usp=sharing

## References

[1] Mascarenhas P , Furtado JM , Almeida SM , Ferraz ME , Ferraz FP , Oliveira P . Pediatric Overweight, Fatness and risk for dyslipidemia are related to diet: a cross-sectional study in 9-year-old children. Nutrients. 2023;15:329. 10.3390/nu15020329. 36678200 PMC9865454

[2] Weihrauch-Blüher S , Schwarz P , Klusmann J-H. Childhood obesity: increased risk for cardiometabolic disease and cancer in adulthood. Metabolism. 2019;92:147–152. 10.1016/j.metabol.2018.12.001. 30529454

[3] Zhang R , Yu X , Yu Y , et al. Family food environments and their association with primary and secondary students’ food consumption in Beijing, China: a cross-sectional study. Nutrients. 2022;14:1970. 10.3390/nu14091970. 35565937 PMC9105134

[4] Mahmood L , Flores-Barrantes P , Moreno LA , Manios Y , Gonzalez-Gil EM. The influence of parental dietary behaviors and practices on children’s eating habits. Nutrients. 2021;13:1138. 10.3390/nu13041138. 33808337 PMC8067332

[5] Bhutta ZA , Ahmed T , Black RE , et al. What works? Interventions for maternal and child undernutrition and survival. The Lancet. 2008;371:417–440. 10.1016/S0140-6736(07)61693-6. 18206226

[6] Hu J , Aris IM , Lin P-ID , et al. Longitudinal associations of modifiable risk factors in the first 1000 days with weight status and metabolic risk in early adolescence. Am J Clin Nutr. 2021;113:113–122. 10.1093/ajcn/nqaa297. 33184628 PMC7779210

[7] Black RE , Liu L , Hartwig FP , et al. Health and development from preconception to 20 years of age and human capital. The Lancet. 2022;399:1730–1740. 10.1016/S0140-6736(21)02533-2. PMC9061873 35489357

[8] Christifano D. Early life nutrition and the developing brain. J Fam Pract. 2023;72:267. 10.12788/jfp.0619. 37549422

[9] Schwarzenberg SJ , Georgieff MK , Daniels S , et al. Advocacy for improving nutrition in the first 1000 days to support childhood development and adult health. Pediatrics. 2018;141:e20173716. 10.1542/peds.2017-3716. 29358479

[10] Reis ÁE de M , Teixeira IS , Maia JM , et al. Maternal nutrition and its effects on fetal neurodevelopment. Nutrition. 2024;125:112483. 10.1016/j.nut.2024.112483. 38823254

[11] Onyango S , Kimani-Murage E , Kitsao-Wekulo P , et al. Associations between exclusive breastfeeding duration and children’s developmental outcomes: evidence from Siaya county, Kenya. PLoS One. 2022;17:e0265366. 10.1371/journal.pone.0265366. 35358207 PMC8970373

[12] Diongue O , Diouf A , Ndour PS , et al. Exclusive breastfeeding measured by deuterium-oxide turnover method is associated with motor development in rural senegalese infants. J Nutr. 2023;153:1850–1857. 10.1016/j.tjnut.2023.02.011. 36792033

[13] Lutter CK , Grummer-Strawn L , Rogers L. Complementary feeding of infants and young children 6 to 23 months of age. Nutr Rev. 2021;79:825–846. 10.1093/nutrit/nuaa143. 33684940

[14] McCormick BJ , Richard SA , Caulfield LE , et al. Early life child micronutrient status, maternal reasoning, and a nurturing household environment have persistent influences on child cognitive development at age 5 years: results from MAL-ED. J Nutr. 2019;149:1460–1469. 10.1093/jn/nxz055. 31162601 PMC6686051

[15] Hammersley ML , Buchanan L , Xu H , Wen LM. Early childhood dietary intake and subsequent socioemotional and cognitive school readiness among australian children. Health Education & Behavior. 2022;49:861–870. 10.1177/10901981221096100. 35668635

[16] Fragkou PC , Karaviti D , Zemlin M , Skevaki C. Impact of early life nutrition on children’s immune system and noncommunicable diseases through its effects on the bacterial microbiome, virome and mycobiome. Front Immunol. 2021;12:644269. 10.3389/fimmu.2021.644269. 33815397 PMC8012492

[17] Naghavi M , Ong KL , Aali A , et al. Global burden of 288 causes of death and life expectancy decomposition in 204 countries and territories and 811 subnational locations, 1990–2021: a systematic analysis for the Global Burden of Disease Study 2021. The Lancet. 2024;403:2100–2132. 10.1016/S0140-6736(24)00367-2. PMC11126520 38582094

[18] Teles LMR , de Oliveira AS , Campos FC , et al. Development and validating an educational booklet for childbirth companions. Revista Da Escola de Enfermagem Da USP. 2014;48:977–984. 10.1590/S0080-623420140000700003. 25626495

[19] Leung MM , Tripicchio G , Agaronov A , Hou N. Manga comic influences snack selection in black and hispanic New York City youth. J Nutr Educ Behav. 2014;46:142–147. 10.1016/j.jneb.2013.11.004. 24433817

[20] Leung MM , Green MC , Tate DF , Cai J , Wyka K , Ammerman AS. *Fight for Your Right to Fruit* : psychosocial outcomes of a *manga* comic promoting fruit consumption in middle-school youth. Health Commun. 2017;32:533–540. 10.1080/10410236.2016.1211074. 27540773

[21] De Rosso S , Ducrot P , Chabanet C , Nicklaus S , Schwartz C. Increasing parental knowledge about child feeding: evaluation of the effect of public health policy communication media in France. Front Public Health. 2022;10:782620. 10.3389/fpubh.2022.782620. 35284356 PMC8907573

[22] Gonçalves S , Ferreira R , Conceição EM , et al. The impact of exposure to cartoons promoting healthy eating on children’s food preferences and choices. J Nutr Educ Behav. 2018;50:451–457. 10.1016/j.jneb.2017.12.015. 29478953

[23] Fonseca LG , Bertolin MNT , Gubert MB , da Silva EF. Effects of a nutritional intervention using pictorial representations for promoting knowledge and practices of healthy eating among Brazilian adolescents. PLoS One. 2019;14:e0213277. 10.1371/journal.pone.0213277. 30856205 PMC6411163

[24] Benevides JL , Coutinho JFV , Pascoal LC , et al. Development and validation of educational technology for venous ulcer care. Revista Da Escola de Enfermagem Da USP. 2016;50:309–316. 10.1590/S0080-623420160000200018. 27384212

[25] de Medeiros CSP , Zanin L , Sperandio M , de Souza Fonseca Silva A , Flório FM. Validation of an educational comic book to guide conducts in situations of dental trauma. Dental Traumatology. 2024;40:161–170. 10.1111/edt.12901. 37881116

[26] Flório FM , Rached EA , Victorelli G , Silva A de SF , Arsati YB de OL . Development and validation of an educational comic book for guidance on the safe use of fluoride toothpaste by children. Pesqui Bras Odontopediatria Clin Integr. 2023;23:e220060. 10.1590/pboci.2023.027.

[27] Martins MC , Veras JEGLF , Uchoa JL , Pinheiro PN da C , Vieira NF da C , Ximenes LB . Segurança alimentar e uso de alimentos regionais: validação de um álbum seriado. Revista Da Escola de Enfermagem Da USP. 2012;46:1354–1361. 10.1590/S0080-62342012000600011. 23380778

[28] Partelli ANM , Cabral IE. Histórias sobre álcool em comunidade quilombola: metodologia participativa de criação-validação de quadrinhos por adolescentes. *Texto & Contexto – Enfermagem*. 2018;26. 10.1590/0104-07072017002820017.

[29] Sridhar A , Friedman S , Grotts JF , Michael B. Effect of theory-based contraception comics on subjective contraceptive knowledge: a pilot study. Contraception. 2019;99:368–372. 10.1016/j.contraception.2019.02.010. 30878456

[30] Wang JL , Acevedo N , Sadler GR. Using comics to promote colorectal cancer screening in the asian american and pacific islander communities. Journal of Cancer Education. 2018;33:1263–1269. 10.1007/s13187-017-1241-4. 28646456

[31] Joshi A , Hillwig-Garcia J , Joshi M , et al. Comics as an educational tool on a clinical clerkship. Academic Psychiatry. 2019;43:290–293. 10.1007/s40596-018-1016-1. 30607894

[32] Morel M , Peruzzo N , Juele AR , Amarelle V. Comics as an educational resource to teach microbiology in the classroom. J Microbiol Biol Educ. 2019;20: 10–1128. 10.1128/jmbe.v20i1.1681. PMC6508913 31160941

[33] Sociedade Brasileira de Pediatria. DEPARTAMENTO DE NUTROLOGIA ALIMENTAÇÃO MANUAL DE DA INFÂNCIA À ADOLESCÊNCIA 4 a EDIÇÃO REVISADA E AMPLIADA. 2018.

[34] Ministério da Saúde. GUIA ALIMENTAR PARA CRIANÇAS BRASILEIRAS MENORES DE 2 ANOS. Brasília: 2019.

[35] Fisk CM , Crozier SR , Inskip HM , Godfrey KM , Cooper C , Robinson SM. Influences on the quality of young children’s diets: the importance of maternal food choices. British Journal of Nutrition. 2011;105:287–296. 10.1017/S0007114510003302. 20807465

[36] Ouyang J , Cai W , Wu P , et al. Association between dietary patterns during pregnancy and children’s neurodevelopment: a birth cohort study. Nutrients. 2024;16:1530. 10.3390/nu16101530. 38794768 PMC11123670

[37] Hossain S , Mihrshahi S. Exclusive breastfeeding and childhood morbidity: a narrative review. Int J Environ Res Public Health. 2022;19:14804. 10.3390/ijerph192214804. 36429518 PMC9691199

[38] Onyango S , Kimani-Murage E , Kitsao-Wekulo P , et al. Associations between exclusive breastfeeding duration and children’s developmental outcomes: evidence from Siaya county, Kenya. PLoS One. 2022;17:e0265366. 10.1371/journal.pone.0265366. 35358207 PMC8970373

[39] Snetselaar LG , de Jesus JM , DeSilva DM , Stoody EE. Dietary guidelines for Americans, 2020–2025. Nutr Today. 2021;56:287–295. 10.1097/NT.0000000000000512. 34987271 PMC8713704

[40] Soriano VX , Ciciulla D , Gell G , et al. Complementary and allergenic food introduction in infants: an umbrella review. Pediatrics. 2023;151:e2022058380. 10.1542/peds.2022-058380. 36704902

[41] Khoury N , Martínez MÁ , Garcidueñas-Fimbres TE , et al. Ultraprocessed food consumption and cardiometabolic risk factors in children. JAMA Netw Open. 2024;7:e2411852. 10.1001/jamanetworkopen.2024.11852. 38758555 PMC11102022

[42] Liu J , Steele EM , Li Y , et al. Consumption of ultraprocessed foods and diet quality among U.S. children and adults. Am J Prev Med. 2022;62:252–264. 10.1016/j.amepre.2021.08.014. 34753645 PMC9384846

[43] Petridi E , Karatzi K , Magriplis E , Charidemou E , Philippou E , Zampelas A. The impact of ultra-processed foods on obesity and cardiometabolic comorbidities in children and adolescents: a systematic review. Nutr Rev. 2024;82:913–928. 10.1093/nutrit/nuad095. 37550263

[44] Correa-Burrows P , Rodríguez Y , Blanco E , Gahagan S , Burrows R. Snacking quality is associated with secondary school academic achievement and the intention to enroll in higher education: a cross-sectional study in adolescents from Santiago, Chile. Nutrients. 2017;9:433. 10.3390/nu9050433. 28448455 PMC5452163

[45] Jamaluddine Z , Akik C , Safadi G , et al. Does a school snack make a difference? An evaluation of the world food programme emergency school feeding programme in Lebanon among Lebanese and Syrian refugee children. Public Health Nutr. 2022;25:1678–1690. 10.1017/S1368980022000362. 35152929 PMC9991733

[46] Alexandre NMC , Coluci MZO. Validade de conteúdo nos processos de construção e adaptação de instrumentos de medidas. Cien Saude Colet. 2011;16:3061–3068. 10.1590/S1413-81232011000800006. 21808894

[47] da Rosa BVC , Girardon-Perlini NMO , Gamboa NSG , Nietsche EA , Beuter M , Dalmolin A. Development and validation of audiovisual educational technology for families and people with colostomy by cancer. Texto e Contexto Enfermagem. 2019;28:e20180053. 10.1590/1980-265X-TCE-2018-0053.

[48] Benevides JL , Coutinho JFV , Pascoal LC , et al. Development and validation of educational technology for venous ulcer care. Revista Da Escola de Enfermagem Da USP. 2016;50:309–316. 10.1590/S0080-623420160000200018. 27384212

[49] Blumfield M , Mayr H , De Vlieger N , et al. Should we ‘eat a rainbow’? An umbrella review of the health effects of colorful bioactive pigments in fruits and vegetables. Molecules. 2022;27:4061. 10.3390/molecules27134061. 35807307 PMC9268388

[50] Vinitchagoon T , Hennessy E , Zhang FF , et al. A dietary pattern with more fruits and vegetables in children of mothers who immigrated to the United States from Latin America is associated with healthful nutrient intake and weight status. J Acad Nutr Diet. 2024;124:947–956. 10.1016/j.jand.2024.04.005. 38609016

[51] Meek JY , Noble L. Policy statement: breastfeeding and the use of human milk. Pediatrics. 2022;150:e2022057988. 10.1542/peds.2022-057988. 35921640

[52] Nigatu D , Azage M , Motbainor A. Effect of exclusive breastfeeding cessation time on childhood morbidity and adverse nutritional outcomes in Ethiopia: analysis of the demographic and health surveys. PLoS One. 2019;14:e0223379. 10.1371/journal.pone.0223379. 31577821 PMC6774524

[53] Chiang K V. , Hamner HC , Li R , Perrine CG. Timing of introduction of complementary foods – United States, 2016–2018. MMWR Morb Mortal Wkly Rep. 2023;69:1969–1973. 10.15585/mmwr.mm6953a1. 37498788 PMC13183349

[54] Roe LS , Meengs JS , Birch LL , Rolls BJ. Serving a variety of vegetables and fruit as a snack increased intake in preschool children. Am J Clin Nutr. 2013;98:693–699. 10.3945/ajcn.113.062901. 23902783 PMC3743731

[55] Jansen E , Thapaliya G , Beauchemin J , D’Sa V , Deoni S , Carnell S. The development of appetite: tracking and age-related differences in appetitive traits in childhood. Nutrients. 2023;15:1377. 10.3390/nu15061377. 36986108 PMC10056659

[56] Beets MW , Tilley F , Kim Y , Webster C. Nutritional policies and standards for snacks served in after-school programmes: a review. Public Health Nutr. 2011;14:1882–1890. 10.1017/S1368980011001145. 21729480

[57] Lopes MV de O , Silva VM da , de Araujo TL . Validação de diagnósticos de enfermagem: desafios e alternativas. Rev Bras Enferm. 2013;66:649–655. 10.1590/S0034-71672013000500002. 24217746

[58] Yusoff MSB. ABC of content validation and content validity index calculation. Education in Medicine Journal. 2019;11:49–54. 10.21315/eimj2019.11.2.6.

[59] Mioramalala SA , Bruand P-E , Ratsimbasoa A , et al. Effects of an educational comic book on epilepsy-related knowledge, attitudes and practices among schoolchildren in Madagascar. Epilepsy Res. 2021;176:106737. 10.1016/j.eplepsyres.2021.106737. 34419769

[60] Kim JH , Lee E , Ha EK , et al. Infant feeding pattern clusters are associated with childhood health outcomes. Nutrients. 2023;15:3065. 10.3390/nu15133065. 37447391 PMC10346276

[61] Mertens A , Benjamin-Chung J , Colford JM , et al. Causes and consequences of child growth faltering in low-resource settings. Nature. 2023;621:568–576. 10.1038/s41586-023-06501-x. 37704722 PMC10511328

[62] Bander A , Murphy-Alford AJ , Owino VO , et al. Childhood BMI and other measures of body composition as a predictor of cardiometabolic non-communicable diseases in adulthood: a systematic review. Public Health Nutr. 2023;26:323–350. 10.1017/S136898002200235X. 36274635 PMC13076086

[63] Moore AM , Fisher JO , Burgess B , Morris KS , Croce CM , Kong KL. Caregiver feeding decisions and sociodemographic characteristics are associated with snack food intake during infancy and toddlerhood. Appetite. 2023;186:106551. 10.1016/j.appet.2023.106551. 37024055 PMC10213156

[64] Almeida C , Azevedo J , Gregório MJ , Barros R , Severo M , Padrão P. Parental practices, preferences, skills and attitudes on food consumption of pre-school children: results from nutriscience project. PLoS One. 2021;16:e0251620. 10.1371/journal.pone.0251620. 34033667 PMC8148319

[65] Mäkelä I , Koivuniemi E , Vahlberg T , Raats MM , Laitinen K. Self-reported parental healthy dietary behavior relates to views on child feeding and health and diet quality. Nutrients. 2023;15:1024. 10.3390/nu15041024. 36839382 PMC9959008

[66] Jilani HS , Pohlabeln H , Buchecker K , et al. Association between parental consumer attitudes with their children’s sensory taste preferences as well as their food choice. PLoS One. 2018;13:e0200413. 10.1371/journal.pone.0200413. 30067786 PMC6070197

[67] Romanos-Nanclares A , Zazpe I , Santiago S , Marín L , Rico-Campà A , Martín-Calvo N. Influence of parental healthy-eating attitudes and nutritional knowledge on nutritional adequacy and diet quality among preschoolers: the SENDO project. Nutrients. 2018;10:1875. 10.3390/nu10121875. 30513857 PMC6316633

[68] De Rosso S , Ducrot P , Chabanet C , Nicklaus S , Schwartz C. Increasing parental knowledge about child feeding: evaluation of the effect of public health policy communication media in France. Front Public Health. 2022;10:782620. 10.3389/fpubh.2022.782620. 35284356 PMC8907573

[69] Sirasa F , Mitchell LJ , Rigby R , Harris N. Family and community factors shaping the eating behaviour of preschool-aged children in low and middle-income countries: a systematic review of interventions. Prev Med (Baltim). 2019;129:105827. 10.1016/j.ypmed.2019.105827. 31476337

[70] Serasinghe N , Vepsäläinen H , Lehto R , et al. Associations between socioeconomic status, home food availability, parental role-modeling, and children’s fruit and vegetable consumption: a mediation analysis. BMC Public Health. 2023;23:1037. 10.1186/s12889-023-15879-2. 37259139 PMC10233887

[71] Moura A , Masquio D. A influência da escolaridade na percepção sobre alimentos considerados saudáveis. Revista de Educação Popular. 2014;13:82–94. 10.14393/REP-v13n12014-art07.

[72] Noiman A , Lee S , Marks K , Grap M , Dooyema C , Hamner H. Factors associated with daily fruit and vegetable intakes among children aged 1–5 years in the United States. Nutrients. 2024;16:751. 10.3390/nu16050751. 38474879 PMC10935013

[73] Guedes JRD , Höfelmann DA , Madruga FP , et al. Associated factors with dietary patterns among children under 2 years of age: a study in childcare centres and homes of South Brazil. J Nutr Sci. 2021;10:e37. 10.1017/jns.2021.26. 35401975 PMC8965686

[74] World Health Organization. Report of the Commission on Ending Childhood Obesity. Implementation plan: executive summary. Geneva 2017.

[75] Juharji H , Albalawi K , Aldwaighri M , et al. Impact of breastfeeding on low birthweight infants, weight disorders in infants, and child development. *Cureus*. 2022. 10.7759/cureus.32894. PMC9870598 36699796

[76] Murray RD. 100% fruit juice in child and adolescent dietary patterns. J Am Coll Nutr. 2020;39:122–127. 10.1080/07315724.2019.1615013. 31318659

[77] O’Neil CE , Nicklas TA , Rampersaud GC , Fulgoni VL. One hundred percent orange juice consumption is associated with better diet quality, improved nutrient adequacy, and no increased risk for overweight/obesity in children. Nutrition Research. 2011;31:673–682. 10.1016/j.nutres.2011.09.002. 22024491

[78] Styne DM , Arslanian SA , Connor EL , et al. Pediatric obesity – assessment, treatment, and prevention: an endocrine society clinical practice guideline. J Clin Endocrinol Metab. 2017;102:709–757. 10.1210/jc.2016-2573. 28359099 PMC6283429

[79] Cifelli CJ , Agarwal S , Fulgoni VL. Association of yogurt consumption with nutrient intakes, nutrient adequacy, and diet quality in American children and adults. Nutrients. 2020;12:3435. 10.3390/nu12113435. 33182430 PMC7696083

[80] Fiore G , Di Profio E , Sculati M , Verduci E , Zuccotti GV. Health effects of yogurt consumption during paediatric age: a narrative review. Int J Food Sci Nutr. 2022;73:738–759. 10.1080/09637486.2022.2065467. 35450518

[81] Moore JB , Horti A , Fielding BA. Evaluation of the nutrient content of yogurts: a comprehensive survey of yogurt products in the major UK supermarkets. BMJ Open. 2018;8:e021387. 10.1136/bmjopen-2017-021387. PMC6144340 30228100

[82] Bechtlufft RP , Costa BLD. Determinantes da desigualdade salarial entre as carreiras do governo de Minas Gerais. Revista de Administração Pública. 2021;55:836–860. 10.1590/0034-761220200879.

[83] Cunha R , Dimenstein M , Dantas C. Desigualdades de gênero por área de conhecimento na ciência brasileira: panorama das bolsistas PQ/CNPq. Saúde Em Debate. 2021;45:83–97. 10.1590/0103-11042021e107.

[84] Benenson JF . The overloaded mother. Arch Sex Behav. 2022;51:3257–3262. 10.1007/s10508-021-01983-0. 33768476

[85] Vieira DA dos S , Castro MA , Fisberg M , Fisberg RM . Nutritional quality of dietary patterns of children: are there differences inside and outside school? J Pediatr (Rio J). 2017;93:47–57. 10.1016/j.jped.2016.03.008. 27362785

[86] Sezer FE , Alpat Yavaş İ , Saleki N , Bakırhan H , Pehlivan M. Diet quality and snack preferences of Turkish adolescents in private and public schools. Front Public Health. 2024;12:1365355. 10.3389/fpubh.2024.1365355. 38496396 PMC10940538

[87] Smith A , Li Y , Du T. Unhealthy after school snacks: socioeconomic disparities of food environments around public and private schools in the United States. Papers in Applied Geography. 2022;8:50–60. 10.1080/23754931.2021.1943499.

[88] Ahrendt Bjerregaard A , Halldorsson TI , Tetens I , Frodi Olsen S. Mother’s dietary quality during pregnancy and offspring’s dietary quality in adolescence: follow-up from a national birth cohort study of 19,582 mother–offspring pairs. PLoS Med. 2019;16:e1002911. 10.1371/journal.pmed.1002911. 31513597 PMC6742222

[89] Murphy MM , Santos-Calderón LA. Long term links between maternal diet during pregnancy and offspring health. *Pediatr Res*. 2024. 10.1038/s41390-024-03726-y. 39543401

